# Supplementary material for: HMGB1 coordinates SASP‐related chromatin folding and RNA homeostasis on the path to senescence
Source: Mol Syst Biol. 2021 Jun 24;17(6):e9760. doi: 10.15252/msb.20209760 (PMC8224457; doi:10.15252/msb.20209760)
Supplement: Supplementary file 2 — Appendix [file MSB-17-e9760-s007.pdf]

Expanded View

HMGB1 coordinates SASP-related chromatin folding and RNA homeostasis on the path to senescence

Konstantinos Sofiadis[1,#], Natasa Josipovic[1,#], Milos Nikolic[2,#], Yulia Kargapolova[2,6], Nadine Übelmesser[1], Ilia Varamogianni[1], Anne Zirkel[2], Ioanna Papadionysiou[1], Gary Loughran[3], James Keane[3,4], Audrey Michel[3], Eduardo G. Gusmao[1], Christian Becker[5], Janine Altmüller[5], Theodore Georgomanolis[2,$], Athanasia Mizi[1] & Argyris Papantonis[1,2,*]

[1] Institute of Pathology, University Medical Center Göttingen, 37075 Göttingen, Germany
[2] Center for Molecular Medicine Cologne, University of Cologne, 50931 Cologne, Germany
[3] Ribomaps, Western Gateway Building, T12 XF62 Cork, Ireland
[4] Cork Institute of Technology, T12 P928 Cork, Ireland
[5] Cologne Center for Genomics, University of Cologne, 50931 Cologne, Germany
[6] Present address: Heart Center, University Hospital Cologne, 50937 Cologne, Germany
[#] These authors contributed equally to this work

*Corresponding author: A.P.; Tel.: +49 551 39 65734, Email: argyris.papantonis@med.uni-goettingen.de

Contents

– This Appendix contains **Figures S1-S6**.

`

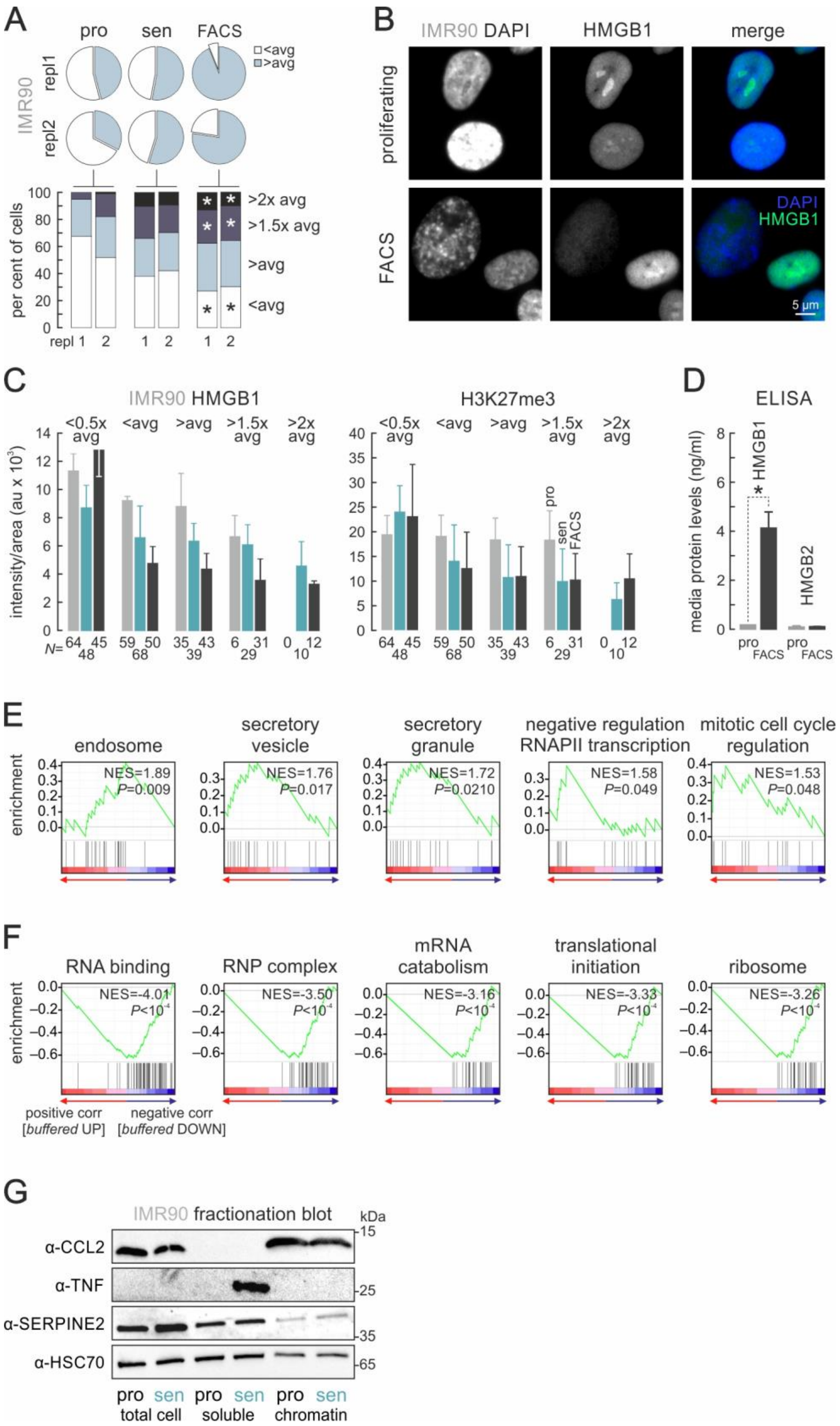
A
pro
sen
FACS
<avg
>avg
IMR90
repl1
repl2
per cent of cells
>2x avg
>1.5x avg
>avg
<avg
repl 1 2 1 2 1 2
B
IMR90 DAPI
HMGB1
merge
proliferating
FACS
DAPI
HMGB1
5 µm
C
IMR90 HMGB1
H3K27me3
<0.5x avg
<avg
>avg
>1.5x avg
>2x avg
intensity/area (au x 10³)
pro
sen
FACS
N=
D
ELISA
media protein levels (ng/ml)
HMGB1
HMGB2
pro
FACS
E
endosome
secretory vesicle
secretory granule
negative regulation RNAPII transcription
mitotic cell cycle regulation
enrichment
NES=1.89 P=0.009
NES=1.76 P=0.017
NES=1.72 P=0.0210
NES=1.58 P=0.049
NES=1.53 P=0.048
F
RNA binding
RNP complex
mRNA catabolism
translational initiation
ribosome
NES=-4.01 P<10⁻⁴
NES=-3.50 P<10⁻⁴
NES=-3.16 P<10⁻⁴
NES=-3.33 P<10⁻⁴
NES=-3.26 P<10⁻⁴
positive corr [buffered UP]
negative corr [buffered DOWN]
G
IMR90 fractionation blot
kDa
α-CCL2
α-TNF
α-SERPINE2
α-HSC70
15
25
35
65
pro sen pro sen pro sen
total cell
soluble
chromatin

Figure S1 HMGB1 is depleted from enlarged nuclei and accompanied by gene expression changes.

A. Pie charts (*top*) of nuclear size in IMR90 populations. Bar graphs (*bottom*) stratify cells according to increasing nuclear size. *: significantly different to proliferating cells, $P<0.05$; Fisher's exact test.
B. Representative images of proliferating (*top row*) and FACS-sorted senescent IMR90 (*bottom row*) immunostained for HMGB1 and counterstained with DAPI. Bar: 5 µm.
C. Bar graphs showing declining HMGB1 (*left*) and H3K27me3 levels (mean ± S.D.; *right*) in proliferating (*grey*), senescent (*green*) and FACS-sorted IMR90 (*black*) stratified according to nuclear size from images like those in panel B. The number of cells analyzed (*N*) is indicated.
D. Bar graphs showing HMGB1/B2 levels (mean ± S.D.) detected in the growth media of proliferating (*grey*) or FACS-sorted IMR90 (*black*). *: $P<0.01$; unpaired two-tailed Student's t-test (N=2).
E. Gene set enrichment analysis of genes translationally upregulated but transcriptionally suppressed in Ribo-seq data. Normalized enrichment scores (NES) and associated *P*-values are shown.
F. As in panel E, but for genes translationally downregulated but transcriptionally upregulated.
G. Chromatin fractionation western blots of exemplary SASP-relevant factors in proliferating and senescent IMR90; CCL2 provides a negative and HSC70 a loading control.

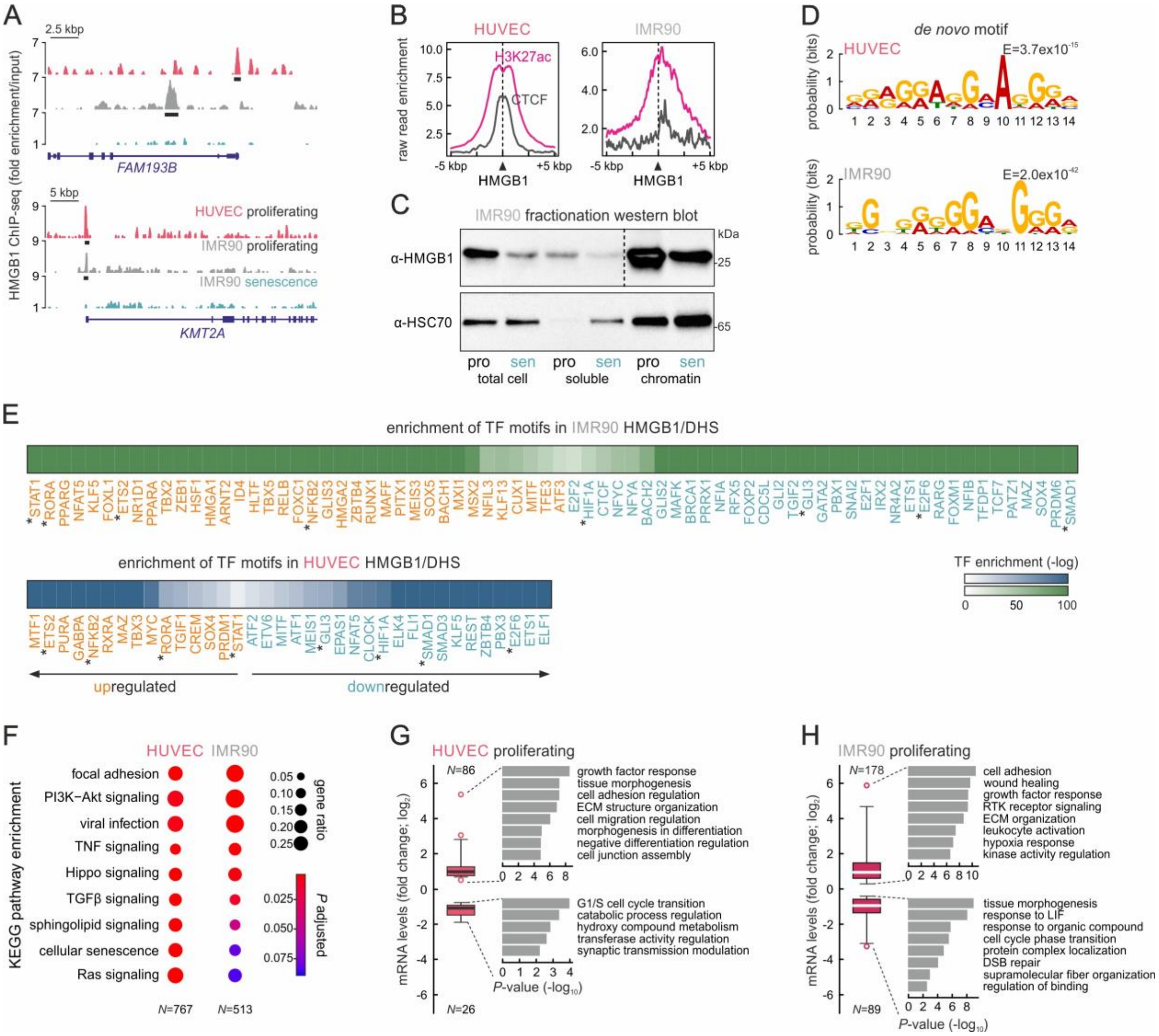

Figure S2 HMGB1 chromatin-binding features in proliferating human cells.

A. Genome browser views showing normalised HMGB1 ChIP-seq signal at the *FAM193B* and *KMT2A* locus from both HUVEC (*pink*) and proliferating/senescent IMR90 (*grey/green*).
B. Line plots showing distribution of H3K27ac (*magenta*) and CTCF ChIP-seq signal (*grey*) in the 10 kbp around HMGB1 peaks from proliferating HUVECs and IMR90.
C. Fractionation western blot in proliferating or senescent IMR90; HSC70 provides a loading control. The chromatin fraction part of the HMGB1 blot (*dashed line*) is shown at a shorter exposure (1.0 sec) than the rest of the blot (5.5 sec; see also Source Data).
D. Logos and associated *E*-values for the most enriched *de novo* motifs under HMGB1 peaks.
E. Heatmap showing most enriched TF binding motifs in the footprints from panel C; up- (>0.6 $\log_2$-fold change; *orange*) or downregulated TF genes (<-0.6 $\log_2$-fold change; *green*) are indicated.
F. Plots KEGG pathways associated with HUVEC and IMR90 HMGB1-bound genes. The number of genes in each cell type (*N*) is indicated.
G. Box plots (*left*) showing significantly up/downregulated genes from proliferating HUVECs with HMGB1 peaks within 20 kbp of their TSS. GO terms associated with these differentially-regulated genes are plotted (*right*). The number of genes in each group (*N*) is indicated.
H. As in panel G, but for HMGB1-bound genes in IMR90.

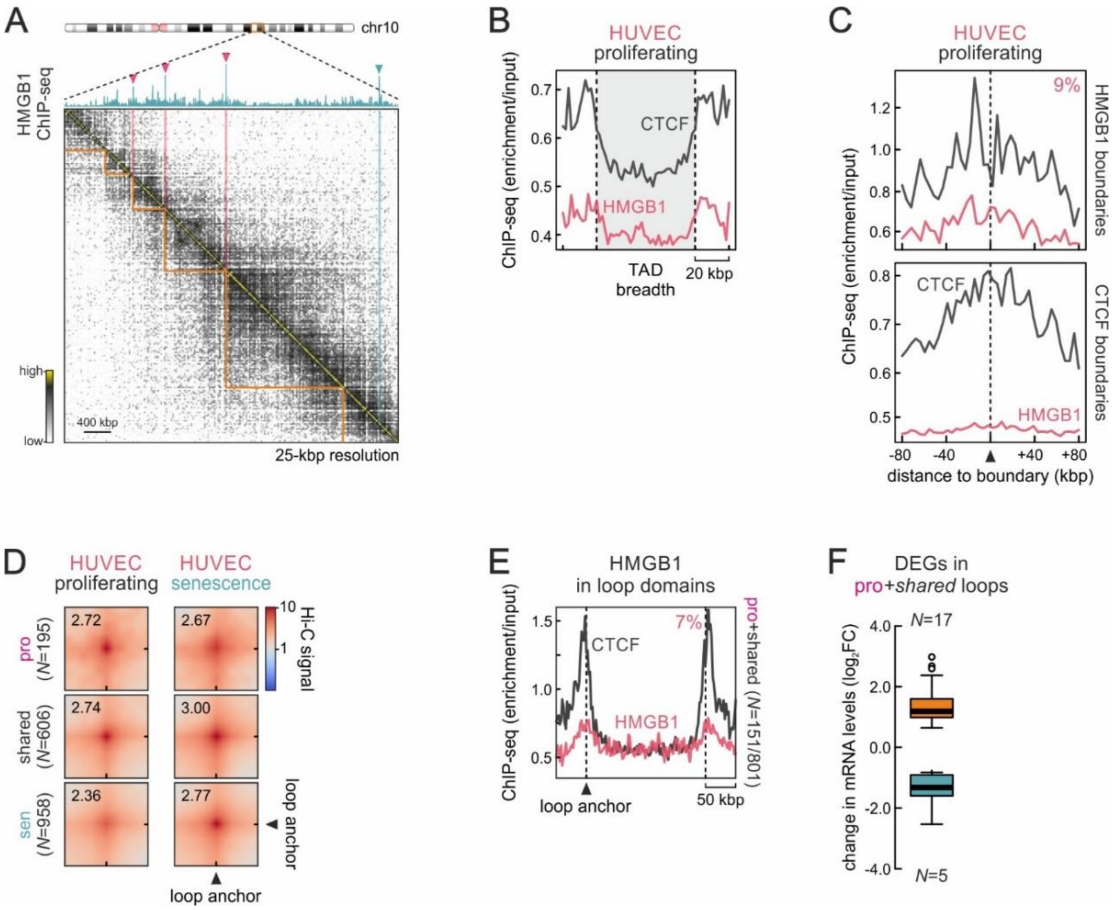

Figure S3 **HMGB1 demarcates TAD and loop domains boundaries in proliferating HUVECs.**

A. Exemplary Hi-C heatmap for a subregion in HUVEC chr10 aligned to HMGB1 ChIP-seq; peaks at TAD boundaries are indicated (*pink arrowheads*).
B. Line plot showing normalised HMGB1 (*green*) and CTCF ChIP-seq signal (*grey*) along all 2888 TADs ±20 kbp from proliferating HUVEC.
C. As in panel B, but in the 160 kbp around HMGB1- (*top*) or CTCF-marked TAD boundaries (*bottom*). The percentage of HMGB1 peaks at boundaries is indicated (*top right*).
D. Aggregate plots showing 20 kbp-resolution Hi-C signal for proliferating (*top*), senescent-specific (*bottom*) or shared loops (*middle*). The number of loops in each category (*N*) is indicated.
E. Line plot showing normalised HMGB1 (*red*) and CTCF ChIP-seq signal (*grey*) along all 801 loop domains TADs ±50 kbp from proliferating HUVEC.
F. Box plot showing significantly up/downregulated HUVEC genes harboured inside the loops from panel E. The number of genes in each group (*N*) is indicated.

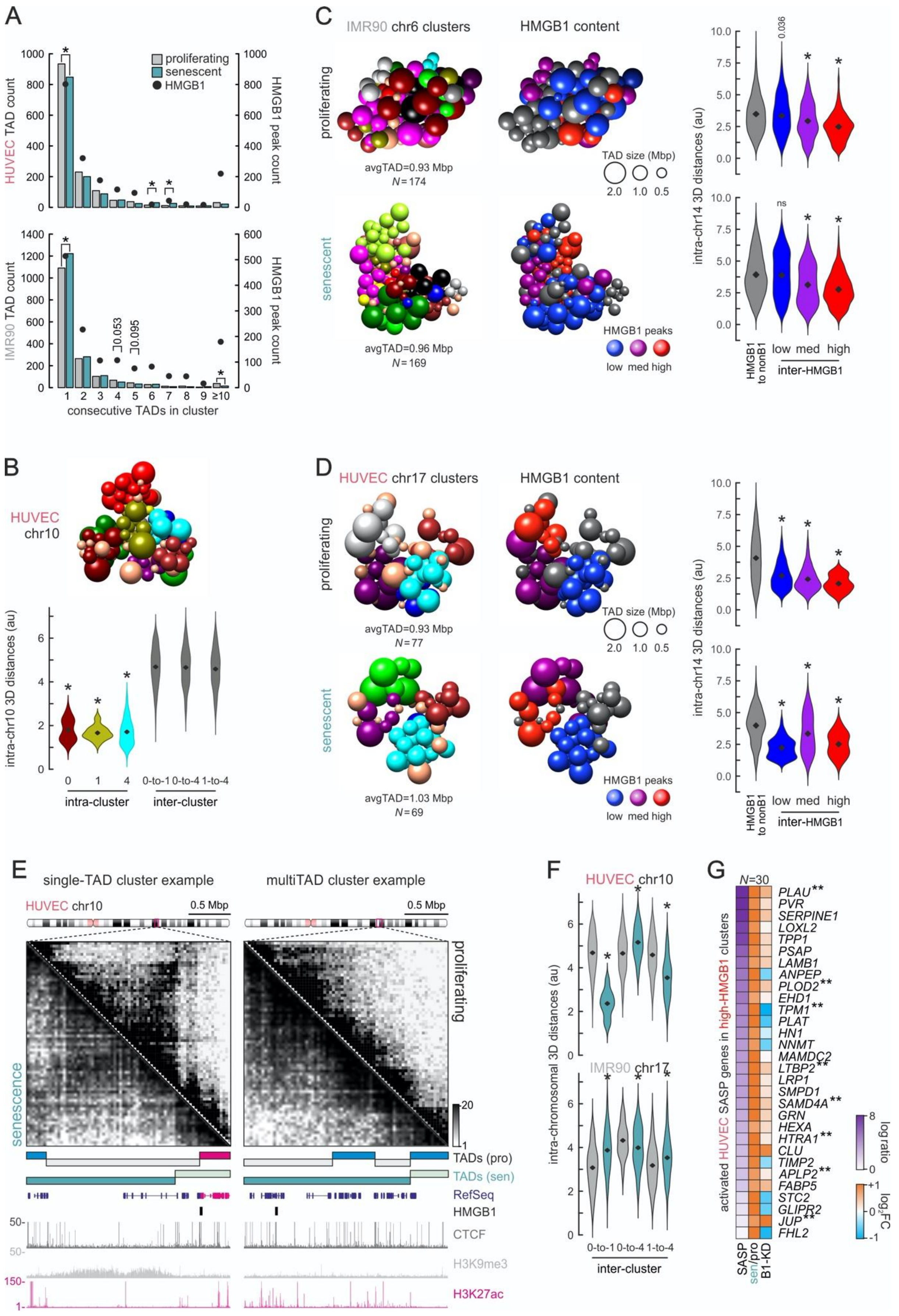

A
B
C
D
E
F
G
HUVEC chr10
IMR90 chr6 clusters
HMGB1 content
HUVEC chr17 clusters
proliferating
senescent
TAD size (Mbp)
HMGB1 peaks
low med high
inter-HMGB1
intra-cluster
inter-cluster
single-TAD cluster example
multiTAD cluster example
TADs (pro)
TADs (sen)
RefSeq
HMGB1
CTCF
H3K9me3
H3K27ac
consecutive TADs in cluster
PLAU**
PVR
SERPINE1
LOXL2
TPP1
PSAP
LAMB1
ANPEP
PLOD2**
EHD1**
TPM1**
PLAT
HN1
NNMT
MAMDC2
LTBP2**
LRP1
SMPD1
SAMD4A**
GRN
HEXA
HTRA1**
CLU
TIMP2
APLP2**
FABP5
STC2
GLIPR2
JUP**
FHL2

Figure S4 HMGB1 demarcates specific TAD subsets in primary human cells.

A. Bar plots showing the number of TADs contained in progressively larger clusters derived using TiLO on proliferating (*grey*) and senescent Hi-C data (*green*) from HUVEC (*top*) and IMR90 (*bottom*). Dots show the absolute number of HMGB1 ChIP-seq peaks in each TiLO cluster category. *: *P*<0.01; Fisher's exact test.
B. Chrom3D visualisation of TAD clustering in chr10 from proliferating HUVEC (*top*); each sphere represents one TAD. Violin plots (*below*) show 3D distances amongst TADs in three randomly-selected clusters (0, 1, and 4) or between TADs from different clusters. *: significantly different to inter-cluster distances, Wilcoxon-Mann-Whitney test.
C. Chrom3D visualisation of TAD clustering in chr17 from proliferating (*top*) and senescent HUVEC (*bottom*). TADs (*spheres*) are coloured by the cluster they belong to (*left*) or according to their HMGB1 ChIP-seq content (*middle*; grey – zero peaks, blue – 1 or 2 peaks, purple – 3 or 4 peaks, red – 5 or more peaks). Violin plots (*right*) show 3D distances amongst TADs in each subgroup or between HMGB1-containing and non-containing TADs. *: significantly different to inter-cluster distances, Wilcoxon-Mann-Whitney test.
D. As in panel E, but for chr6 from proliferating and senescent IMR90.
E. *Left*: Exemplary Hi-C data (40-kbp resolution) around a single-TAD cluster (*magenta*) in HUVEC chr10 aligned to TAD positions, HMGB1 peaks, and ENCODE ChIP-seq. *Right*: Exemplary Hi-C data for TADs forming a multi-TAD cluster in senescent HUVEC according to TiLO.
F. Violin plots showing interchromosomal distances between TADs in different clusters of HUVEC chr10 (*top*) or IMR90 chr17 (*bottom*) based on TiLO/Chrom3D visualisations. *: significantly different to inter-cluster distances, Wilcoxon-Mann-Whitney test.
G. Heatmap showing protein (SASP, from http://www.saspatlas.com/; log)and gene expression levels (log_2FC) of HUVEC SASP-related genes embedded in high-HMGB1 (red) TADs like those in panel E. **: genes bound by HMGB1 in HUVEC ChIP-seq data are more than expected by chance; *P*>0.001, hypergeometric test.

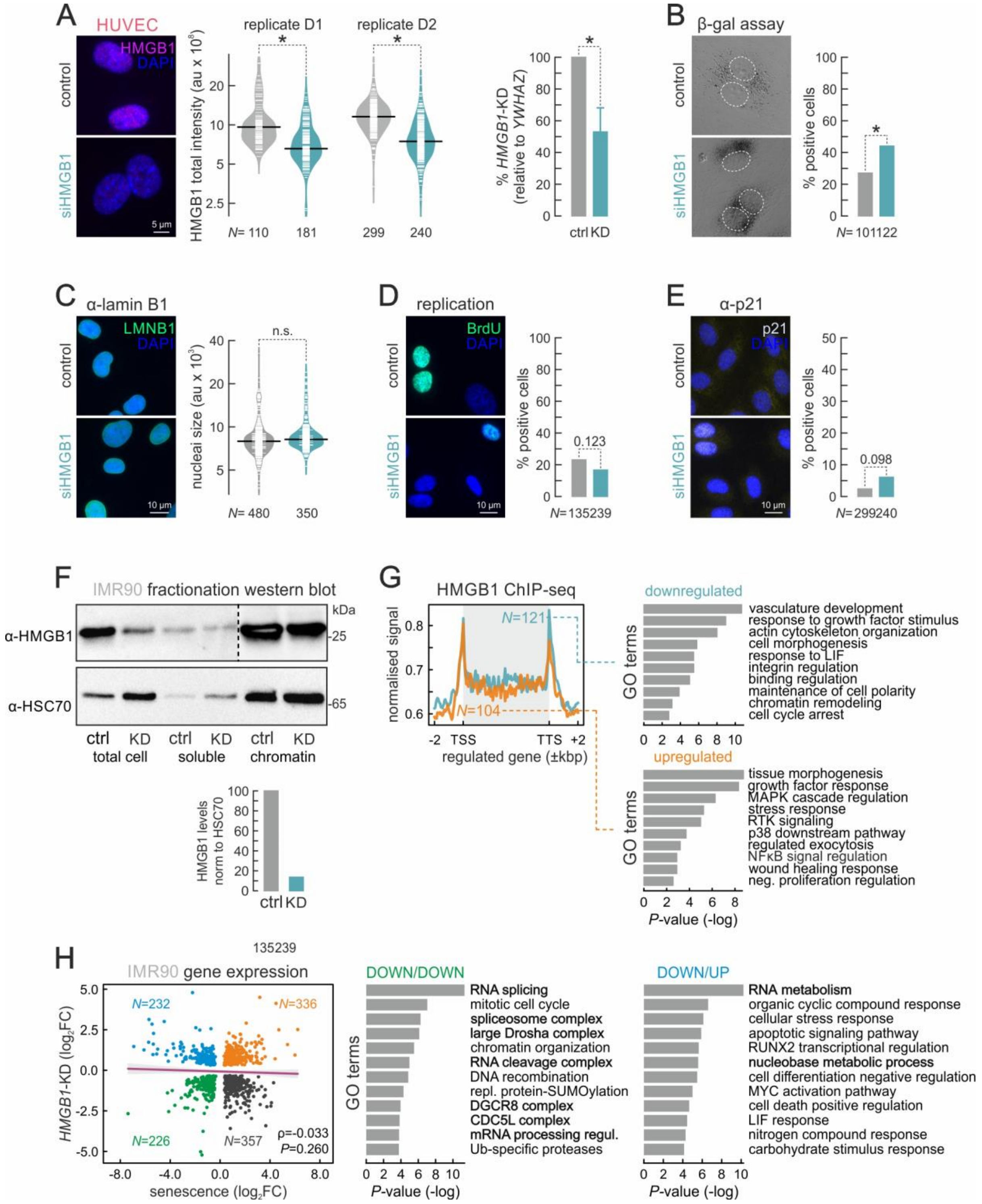

A
HUVEC
HMGB1
DAPI
control
siHMGB1
5 µm
replicate D1
replicate D2
HMGB1 total intensity (au x 10^8)
N= 110
181
299
240
% HMGB1-KD (relative to YWHAZ)
ctrl KD
B
β-gal assay
control
siHMGB1
% positive cells
N= 101122
C
α-lamin B1
LMNB1
DAPI
nuclei size (au x 10^3)
n.s.
N= 480
350
10 µm
D
replication
BrdU
DAPI
% positive cells
0.123
N=135239
E
α-p21
p21
DAPI
% positive cells
0.098
N=299240
F
IMR90 fractionation western blot
α-HMGB1
α-HSC70
kDa
-25
-65
ctrl KD ctrl KD ctrl KD
total cell
soluble
chromatin
HMGB1 levels norm to HSC70
ctrl KD
G
HMGB1 ChIP-seq
normalised signal
N=121
N=104
-2 TSS TTS +2
regulated gene (±kbp)
downregulated
GO terms
vasculature development
response to growth factor stimulus
actin cytoskeleton organization
cell morphogenesis
response to LIF
integrin regulation
binding regulation
maintenance of cell polarity
chromatin remodeling
cell cycle arrest
upregulated
tissue morphogenesis
growth factor response
MAPK cascade regulation
stress response
RTK signaling
p38 downstream pathway
regulated exocytosis
NFκB signal regulation
wound healing response
neg. proliferation regulation
P-value (-log)
135239
H
IMR90 gene expression
HMGB1-KD (log2FC)
senescence (log2FC)
N=232
N=336
N=226
N=357
ρ=-0.033
P=0.260
DOWN/DOWN
RNA splicing
mitotic cell cycle
spliceosome complex
large Drosha complex
chromatin organization
RNA cleavage complex
DNA recombination
repl. protein-SUMOylation
DGCR8 complex
CDC5L complex
mRNA processing regul.
Ub-specific proteases
DOWN/UP
RNA metabolism
organic cyclic compound response
cellular stress response
apoptotic signaling pathway
RUNX2 transcriptional regulation
nucleobase metabolic process
cell differentiation negative regulation
MYC activation pathway
cell death positive regulation
LIF response
nitrogen compound response
carbohydrate stimulus response
GO terms
P-value (-log)

Figure S5 ***HMGB1*-knockdown effects in proliferating HUVECs and IMR90.**

A. Representative immunofluorescence images of HMGB1 (*left*) in siHMGB1-treated and control cells counterstained with DAPI. Bean plots quantify knockdown efficiency (*middle*; *N* is the number of cells analyzed). Bar graphs (*right*) show reduction in *HMGB1* mRNA levels (±S.D.; *N*=2) in knockdown compared to control cells. Bar: 5 µm. *: *P*<0.01; Wilcoxon-Mann-Whitney and unpaired two-tailed Student's t-test for bean and bar plots, respectively.
B. Representative brightfield images of siHMGB1-treated HUVECs showing elevated β-galactosidase activity compared to control cells (*left*); bar graphs quantify this increase (*right*; *N* indicates the number of cells analyzed). *: *P*<0.01; Fisher's exact test.
C. As in panel A, but for LMNB1 levels. Bar: 10 µm. No statistically significant difference (n.s.); Wilcoxon-Mann-Whitney test.
D. As in panel A, but for BrdU incorporation. Bar: 10 µm. *P*=0.123; Fisher's exact test.
E. As in panel A, but for p21 levels. Bar: 10 µm. *P*=0.098; Fisher's exact test.
F. Fractionation western blot (*top*) in control and HMGB1-knockdown IMR90; HSC70 provides a loading control. Bar graphs (*below*) quantify the reduction in chromatin-bound HMGB1 after normalization to the loading control. The chromatin fraction of the HMGB1 blot (*dashed line*) is shown at a shorter exposure (1.0 sec) than the rest of the blot (3.3 sec; see also Source Data).
G. Line plots (*left*) showing normalised HMGB1 ChIP-seq signal along genes up- (*orange*) or downregulated upon *HMGB1*-knockdown in IMR90 (*green*; *N* indicates the number of HMGB1-bound genes). Bar plots (*right*) showing GO terms associated up/downregulated genes and their enrichment *P*-values.
H. Scatter plots (*left*) showing correlation between differentially-expressed genes ($\log_2$FC) upon senescence entry and *HMGB1*-knockdown in IMR90. Pearson's correlation (ρ) and associated *P*-value are shown alongside the number of genes (*N*) in each subgroup. Bar plots (*right*) showing GO terms and enrichment *P*-values associated with genes commonly downregulated ("DOWN/DOWN") or downregulated in senescence but upregulated upon *HMGB1*-knockdown ("DOWN/UP"). Terms relevant to RNA processing are highlighted (*bold*).

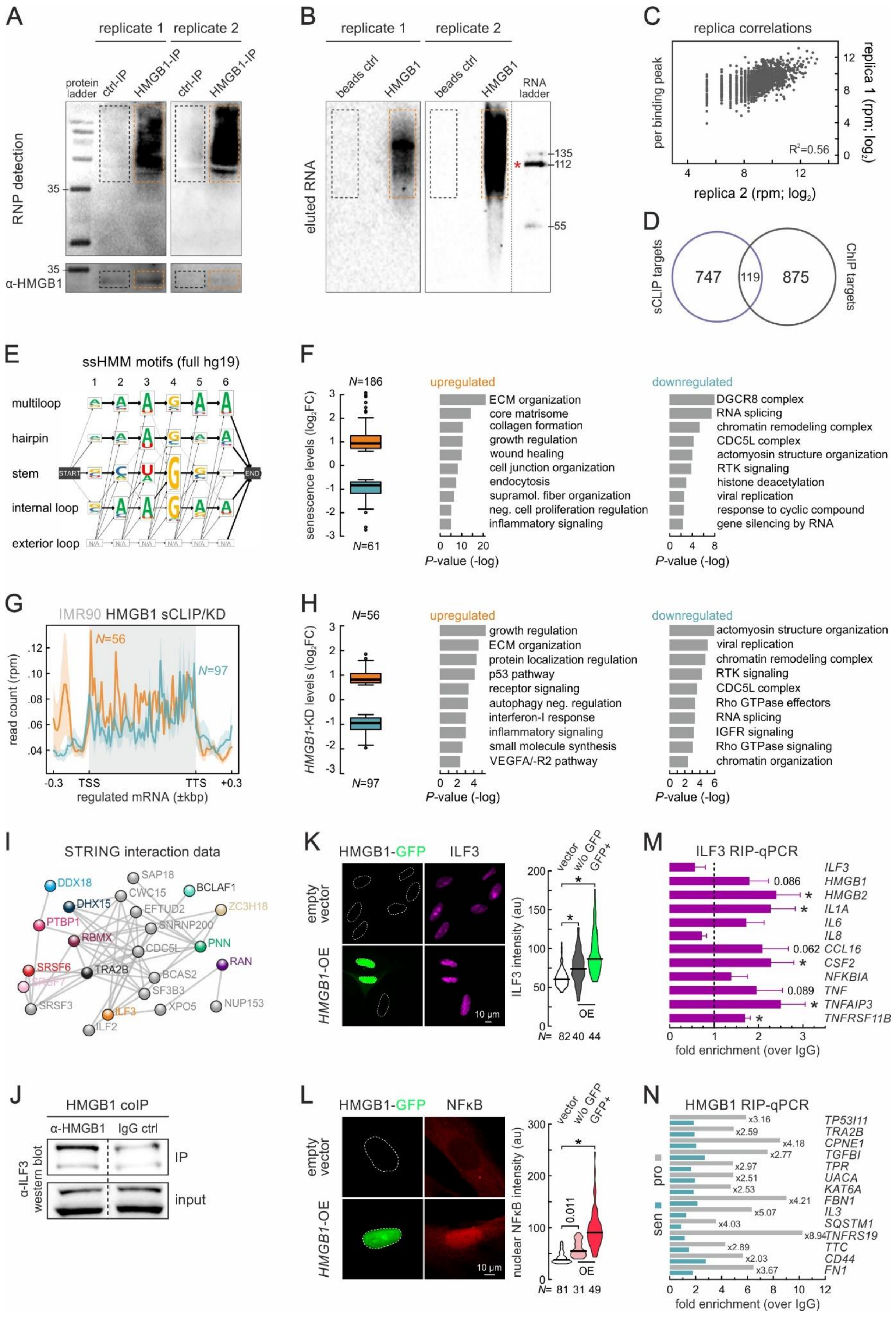
A
replicate 1
replicate 2
protein ladder
ctrl-IP
HMGB1-IP
ctrl-IP
HMGB1-IP
RNP detection
35
35
α-HMGB1
B
replicate 1
replicate 2
beads ctrl
HMGB1
beads ctrl
HMGB1
RNA ladder
eluted RNA
135
112
55
C
replica correlations
per binding peak
replica 1 (rpm; log2)
replica 2 (rpm; log2)
R²=0.56
D
sCLIP targets
747
119
875
ChIP targets
E
ssHMM motifs (full hg19)
multiloop
hairpin
stem
internal loop
exterior loop
START
END
F
N=186
N=61
senescence levels (log2FC)
upregulated
ECM organization
core matrisome
collagen formation
growth regulation
wound healing
cell junction organization
endocytosis
supramol. fiber organization
neg. cell proliferation regulation
inflammatory signaling
P-value (-log)
downregulated
DGCR8 complex
RNA splicing
chromatin remodeling complex
CDC5L complex
actomyosin structure organization
RTK signaling
histone deacetylation
viral replication
response to cyclic compound
gene silencing by RNA
P-value (-log)
G
IMR90 HMGB1 sCLIP/KD
N=56
N=97
read count (rpm)
-0.3
TSS
TTS
+0.3
regulated mRNA (±kbp)
H
N=56
N=97
HMGB1-KD levels (log2FC)
upregulated
growth regulation
ECM organization
protein localization regulation
p53 pathway
receptor signaling
autophagy neg. regulation
interferon-I response
inflammatory signaling
small molecule synthesis
VEGFA/-R2 pathway
P-value (-log)
downregulated
actomyosin structure organization
viral replication
chromatin remodeling complex
RTK signaling
CDC5L complex
Rho GTPase effectors
RNA splicing
IGFR signaling
Rho GTPase signaling
chromatin organization
P-value (-log)
I
STRING interaction data
DDX18
SAP18
BCLAF1
CWC15
DHX15
EFTUD2
ZC3H18
PTBP1
SNRNP200
RBMX
PNN
CDC5L
RAN
SRSF6
TRA2B
SRSF7
BCAS2
SF3B3
SRSF3
ILF3
XPO5
NUP153
ILF2
J
HMGB1 coIP
α-HMGB1
IgG ctrl
α-ILF3 western blot
IP
input
K
HMGB1-GFP
ILF3
empty vector
HMGB1-OE
10 μm
vector
w/o GFP
GFP+
ILF3 intensity (au)
OE
N= 82 40 44
L
HMGB1-GFP
NFκB
empty vector
HMGB1-OE
10 μm
vector
w/o GFP
GFP+
nuclear NFκB intensity (au)
0.011
OE
N= 81 31 49
M
ILF3 RIP-qPCR
ILF3
HMGB1
HMGB2
IL1A
IL6
IL8
CCL16
CSF2
NFKBIA
TNF
TNFAIP3
TNFRSF11B
0.086
0.062
0.089
fold enrichment (over IgG)
N
HMGB1 RIP-qPCR
pro
sen
TP53I11
TRA2B
CPNE1
TGFBI
TPR
UACA
KAT6A
FBN1
IL3
SQSTM1
TNFRS19
TTC
CD44
FN1
x3.16
x2.59
x4.18
x2.77
x2.97
x2.51
x2.53
x4.21
x5.07
x4.03
x8.94
x2.89
x2.03
x3.67
fold enrichment (over IgG)

Figure S6 HMGB1 sCLIP controls and analysis of HMGB1-interacting RBPs.

A. Electrophoretic profiles of control (beads only; *black dotted square*) and HMGB1 IP (*orange dotted rectangle*) probed for RNA (*top*) or HMGB1 (*bottom*) in both sCLIP replicates. The 35-kDa position of the molecular mass ladder is indicated.
B. As in panel A, but for RNA eluted from control (beads only; *black dotted square*) or HMGB1 IP (*orange dotted rectangle*) in both sCLIP replicates. The 112-nt band in the molecular mass ladder (*red star*) corresponds to ~144 pg of RNA.
C. Scatter plot showing correlation of sCLIP data from two independent biological replicates compared per binding peak normalized read count. Spearman correlation values (R^2) are shown.
D. Venn diagram showing overlap of HMGB1 sCLIP and ChIP-seq targets in proliferating IMR90.
E. ssHMM motif analysis of sCLIP data showing sequence probabilities in HMGB1-bound motifs predicted to form different structures using the reference human genome (hg19).
F. Box plots (*left*) showing change ($\log_2$) in mRNAs differentially-regulated upon senescence and bound by HMGB1 in IMR90 sCLIP. The number of mRNAs (*N*) analyzed is indicated. Bar graphs (*right*) showing GO terms associated with each subgroup and their enrichment *P*-values.
G. Line plots showing mean HMGB1 sCLIP signal along mRNAs up- (*orange*) or downregulated (*green*) upon *HMGB1*-knockdown in IMR90. The number of HMGB1-bound mRNAs (*N*) is indicated.
H. As in panel F, but for the up-/downregulated mRNAs from panel F.
I. Experimentally-validated protein-protein interaction network for HMGB1-coimmunoprecipitating RBPs (*colored spheres*) that are also downregulated in senescence. Their secondary interactors (*grey spheres*) from the STRING database are also shown.
J. Western blots showing ILF3 co-immunoprecipitating with HMGB1 (*left*) or IgG (*right*) in IMR90; blots on 10% of input material provide a loading control.
K. Representative images of IMR90 overexpressing HMGB1-GFP and immunostained for ILF3. Outlines of nuclei are indicated (*dashed lines* based on DAPI counterstaining). IMR90 transfected with an empty vector provides a control. Bar: 10 µm. Signal intensities are quantified in the violin plots (*right*). *: significantly different to control; *P*<0.001, Wilcoxon-Mann-Whitney test
L. As in panel K, but immunostained for phospho-NFκB. Bar: 10 µm.
M. Bar graphs showing mean fold-enrichments (over IgG controls ±S.D., *N*=2) of selected mRNAs from ILF3 RIP experiments in proliferating IMR90. *: significantly different to control; *P*<0.05, unpaired two-tailed Student's t-test.
N. As in panel M, but showing mean HMGB1 enrichment on selected mRNA targets in proliferating (*grey*) and senescent IMR90 (*green*). Fold differences between the two states are indicated.
