# Supplementary material for: HMGB1 coordinates SASP‐related chromatin folding and RNA homeostasis on the path to senescence
Source: Mol Syst Biol. 2021 Jun 24;17(6):e9760. doi: 10.15252/msb.20209760 (PMC8224457; doi:10.15252/msb.20209760)
Supplement: Supplementary file 10 — Source Data for Appendix [file MSB-17-e9760-s008.pdf]

Source Data for Figure S2C

α-HMGB1

α-HMGB1

α-HSC70

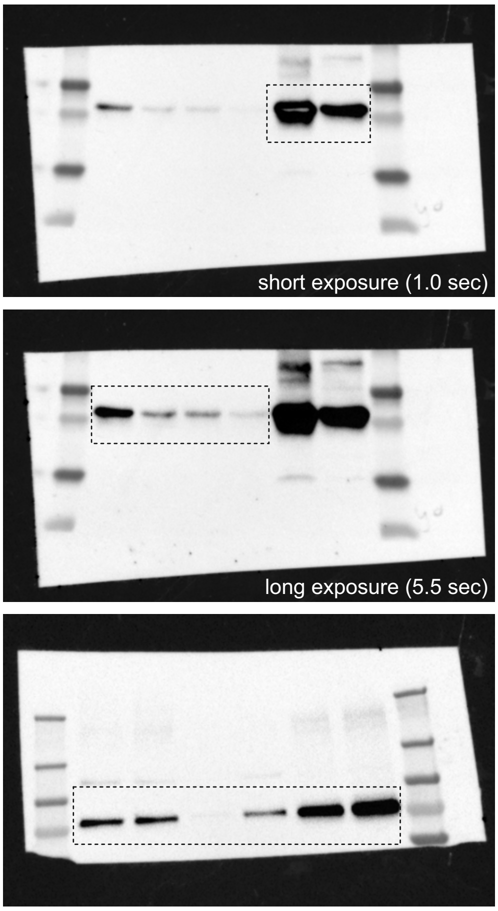

Source Data for Figure S5F

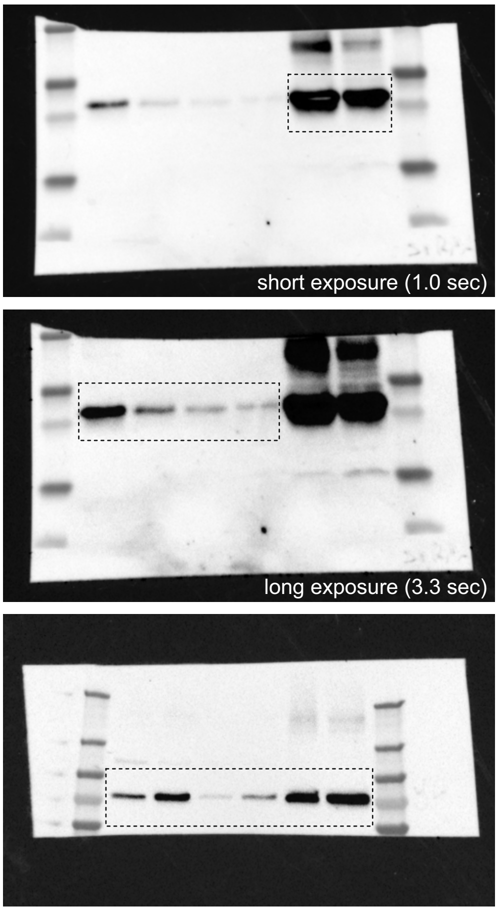

**dashed rectangles indicate regions used in figures
